# Impact of *Sumadhur* intervention on fertility and family planning decision-making norms: a mixed methods study

**DOI:** 10.1186/s12978-023-01619-7

**Published:** 2023-05-25

**Authors:** Ashley Mitchell, Mahesh C. Puri, Minakshi Dahal, Alia Cornell, Ushma D. Upadhyay, Nadia G. Diamond-Smith

**Affiliations:** 1 Institute for Global Health Sciences, University of California, San Francisco, CA USA; 2 Bixby Center for Global Reproductive Health, San Francisco, CA USA; 3 Center for Research On Environment, Health and Population Activities, Kathmandu, Nepal; 4 University of California, Los Angeles, CA USA; 5 Department of Obstetrics, Gynecology, and Reproductive Sciences, University of California, San Francisco, CA USA; 6 Department of Epidemiology and Biostatistics and Institute for Global Health Sciences, University of California, San Francisco, CA USA

**Keywords:** Fertility, Family planning, Intervention, Pregnancy, Intergenerational households, Norms, Gender, Couple dynamics

## Abstract

**Background:**

Mindful of social norms shaping health among women pressured to prove early fertility in Nepal, a bi-national research team developed and piloted a 4-month intervention engaging household triads (newly married women, their husbands, and mothers-in-law) toward advancing gender equity, personal agency, and reproductive health. This study evaluates the impact on family planning and fertility decision-making.

**Methods:**

In 2021, *Sumadhur* was piloted in six villages with 30 household triads (90 participants). Pre/post surveys of all participants were analyzed using paired sample nonparametric tests and in-depth interviews with a subset of 45 participants were transcribed and analyzed thematically.

**Results:**

*Sumadhur* significantly impacted (p < .05) norms related to pregnancy spacing and timing, and sex preference of children, as well as knowledge about family planning benefits, pregnancy prevention methods, and abortion legality. Family planning intent also increased among newly married women. Qualitative findings revealed improved family dynamics and gender equity, and shed light on remaining challenges.

**Conclusions:**

Entrenched social norms surrounding fertility and family planning contrasted with participants’ personal beliefs, highlighting needed community-level shifts to improve reproductive health in Nepal. Engagement of influential community- and family-members is key to improving norms and reproductive health. Additionally, promising interventions such as *Sumadhur* should be scaled up and reassessed.

## Plain language summary

Societal norms are among the key influencers that shape the decisions that people make about their desired family size and the methods they will apply to achieve it. To support women in Nepal, where norms are often layered upon the expectation that women will prove their fertility soon after marriage, a bi-national research team developed and piloted a 4-month intervention, *Sumadhur*, engaging newly married women, their husbands, and mothers-in-law. This study evaluated the impact the *Sumadhur* had on norms, knowledge, and intent related to family planning. From pre- and post-questionnaires, we found norms significantly shifted and knowledge significantly improved among all participant groups as a result of participating in *Sumadhur*. From interviews following the intervention, we found that family dynamics and gender equity also improved despite lingering challenges including unchanged norms about the expected timing of a couple’s first child. Our results confirmed that it is critical to engage influential community and family members in improving norms and supporting women to make decisions about their reproduction. Additionally, promising interventions like *Sumadhur* should be scaled up and re-evaluated.

## Background

Globally, fertility and family planning decision-making is shaped by many intersecting factors including perceived social norms, especially for young, newly married women who are pressured to prove fertility early in marriage. In populations in which these women are acutely disempowered and hold the lowest household status, including within South Asia, this is particularly true [1]. Social norms can act as a barrier or as a facilitator of individual intentions and behavior change [2]. Accordingly, understanding the social norms and expectations surrounding fertility and family planning is critical to support individuals, couples, and families in their decision-making. Historically, in Nepal, married women who have not yet given birth to a child, especially a male child, experience reduced personal agency and autonomy through confinement within their homes and restricted access to healthcare, among other restrictions on diet and behavior [3, 4]. These manifestations of fertility-related social norms are critical to assess as approximately half of newly married women in Nepal become pregnant within one year of marriage [5].

While recent national estimates suggest that around 20% of pregnancies in Nepal are unwanted, local research reports that 34–59% of all pregnancies in Nepal are unintended, varying slightly by geographic region [5, 6]. The 2016 Nepal Demographic and Health Survey suggests that about a quarter of women have an unmet need for contraception, which is disproportionately experienced by communities in the lowest income quintile [5]. Hormonal methods are most commonly provided by the government sector and use is highest among families that already have three or four children, the majority of whom are ages 35 to 44 years old [5]. There is a dearth of data about unmet family planning needs among unmarried women in Nepal as sexual activity is seldom reported by this population [7]. Furthermore, though first-trimester abortion has been generally legal since 2002, and provided free of cost since 2016 at public health facilities, many Nepali women and girls continue to face challenges in exercising their fundamental right to obtain safe abortion services [8, 9]. Among other factors, lack of awareness of the legality of abortion and its availability, location, and costs of services prevents many women from seeking and accessing services [6, 9, 10].

Throughout many communities in Nepal, marriages are often arranged, and family members are intimately involved in the couples’ new life together—with many couples moving into the husband’s family’s home following marriage [11]. Co-residence in this setting often results in shared decision-making at a household level, making family engagement critical in any intervention targeting married women, especially newly married [12]. Prior research has found that household interventions improve women’s livelihood and suggest that women’s agency can be advanced by their families and communities [12, 13]. A recent study in rural Nepal concluded that engaging mothers-in-law, specifically, was critical to improving maternal and child health [14]. Results from a longitudinal study in the neighboring country of India suggested that engaging both women and men led to improved family planning outcomes [15]. We found very few interventions, however, engaging intact household groups in this region or on these topics. The studies analyzing this approach reported promising results, suggesting household engagement was acceptable and effective for improving knowledge and changing behaviors [12, 16, 17].

Group interventions have been found to successfully increase health knowledge in South Asia and remain an important tool for challenging social norms related to fertility and family planning that are coercive or threaten reproductive autonomy [18–20]. Additionally, those that specifically promote gender equity and address gender norms, an important subset of social norms, have been found to positively impact health outcomes among women [21]. Women’s personal agency and autonomy are too often restricted by inequitable gender norms, some of which result in violence and other violations of human rights [22]. In Nepal, women are already disproportionately at an increased risk of experiencing household violence with estimates of intimate partner violence and related abuse ranging from about 25–50% [23–25]. Promisingly, however, research continues to find that women’s empowerment is positively related to contraceptive intention and ever use as well as couple communication about family planning [26]. Positive associations between measures of gender equity, such as sharing childcare responsibilities, and use of contraception have also been found in Nepal, specifically [27].

Despite awareness of the common dynamics of shared decision-making surrounding health, household- and community-level influences on women’s wellbeing and personal agency, very few interventions engage multiple household members together, nor address social norms through a group community-level approach [16, 17]. The Center for Research on Environment Health and Population Activities (CREHPA), Vijaya Development Resource Centre (VDRC-Nepal), and the University of California San Francisco (UCSF) collaborated to develop and pilot an interactive group intervention for newly married households in Nepal. Mindful of the strong influence of perceived societal expectations on knowledge and health-seeking behaviors, the intervention sought to address the individual and social norms that underpin decision-making related to fertility and family planning, and women’s status more broadly (gender norms). The purpose of this study is to evaluate the impact of the intervention on gender equity via norms around family planning and fertility decision-making among newly young married women (aged 18–25 years), their husbands, and their mothers-in-law in six Nepalese villages.

## Methods

### Intervention

Through a community engaged design approach and informed by a longitudinal (2-year) mixed methods study, we collaboratively developed and piloted *Sumadhur* (meaning “Best Relationship)*,* an intervention for newly married households in Nawalparasi District of Nepal [28]. *Sumadhur* is a four-month long, weekly group intervention for triads (wives, husbands, and mothers-in-law) that covers various topics including nutrition, anemia, intrahousehold food allocation, prenatal health and pregnancy care, inequitable gender norms and practices, fertility planning and contraception, and couples and household relationship dynamics. Each session combined educational information with interactive topic-related games and activities that helped build relationships and break social and gender norms. More details about the development and content in the intervention are reported on by Diamond-Smith et al., 2022 [28].

*Sumadhur* was piloted in six villages in Nawalparasi District of Nepal from January through May 2021 in one rural (Palhinandan) and one urban (Sunwal) municipality. Each group had five households, for a total of 15 people per group and 90 people in the intervention: 30 newly married women, 30 husbands (although two husbands emigrated out of the municipality during the intervention period resulting in 28 husband respondents at endline) and 30 mothers-in-law. Trained researchers conducted pre-post surveys with all participants and in-depth qualitative interviews with a subset of 45 participants at endline (all three members from 15 households, at which point the team felt that saturation had been reached). Participants for the qualitative interviews were randomly chosen and were roughly evenly divided between the two municipalities and across the 6 villages. Participants were interviewed by four trained female Nepali researchers in a private location of their choice at a time of their choice and in the local language, Awadhi. Researchers obtained informed, written consent from the participants before data collection. Participants received a small gift in appreciation of their time for the interviews. There was no incentive for participating in the intervention.

The study received ethical approvals from the Nepal Health Research Council (Reg No 385/2016) and the ethics committee of the University of California, San Francisco.

### Study approach and timeline

We used an explanatory sequential mixed methods design, where our analysis of the quantitative survey data informed the refinement of endline in-depth interview guides to collect and analyze qualitative data—facilitating improved understanding about the findings from our survey data [29]. “Baseline” in this study refers to survey data collected in January, 2021 before the start of the four month intervention (*Sumadhur*). “Endline” in this study refers to survey data collected in March and April, 2021 following the intervention. All qualitative interviews for this study were conducted in July, 2021—several months after the intervention due to a COVID-19 surge and subsequent social restrictions.

### Measures

The survey and in-depth interview instruments were co-developed in partnerships with local collaborators and pilot tested with newly married women to test for content validity. Where possible scales and items were used which have been previously validated in the Nepali context.

Survey questions were categorized in alignment with the “Norms on Fertility” and “Norms on Decision-making and agency” within the Family Planning Social Norms Taxonomy developed by Breakthrough ACTION [30]. This taxonomy accounts for both descriptive norms or perceived behaviors and injunctive norms or perceived expectations governing behavior [30]. Categories and sub-categories of the original model were adapted slightly, as intended by its authors, to account for sociocultural nuances specific to our setting and population.

“Norms about fertility” included four questions related to timing, spacing, preferred family size, and sex preference of children (Table 2). Two questions were asked related to timing and spacing, where participants were asked to respond to using a 5-choice Likert scale from “Strongly agree” to “Strongly disagree” to the statements (1) “*It is wrong to use contraceptives or other means to avoid or delay pregnancy before having had at least one birth*.” and (2) *“It is wrong to use contraceptives or other means to avoid or delay pregnancy.”* Two questions were asked related to family size and sex preference, where participants were asked open-ended questions instructing them to quantify their desired number of total children (or grandchildren, if mother-in-law) as well as the number of boys, girls, or either that they desired.

“Norms about decision-making” included three questions related to couple dynamics and household roles for all participants, as well as one question regarding individual intent to use contraceptives for newly married women and husbands (Table 2). Related to couple dynamics and household roles, participants were asked to respond to using a 5-choice Likert scale from “Strongly agree” to “Strongly disagree” to the statements 1) “*Most families you know believe men should make the decision about whether or not their wife uses birth control.*”, 2) “*Most families you know believe husbands should have the final say about when to start trying to have their first child*.”, and 3) “*Most families you know believe husbands should make the final decision about the total number of children they want.”* Related to individual intentions, newly married women and husbands were asked *“*Do you think you will use a contraceptive method to delay or avoid pregnancy at any time in the future?*”* and could respond with “Yes”, “No”, or “I don’t know”.

The analysis of knowledge and behavior included four questions related to the benefits and identification of pregnancy prevention methods and the legality of abortion, as well as one question regarding ever use of contraception for newly married women and husbands and one question regarding pregnancy occurrence, asked only of newly married women (Table 3). Related to benefits, participants were asked the open-ended question “What are the benefits of birth spacing?” and relevant responses including improved maternal and child health and improved economic and educational opportunities, among others, were counted. Of note, while some participants responded with “None” or “Don’t know”, no responses were deemed incorrect. Related to the identification of methods, participants were asked to list “*some of the temporary family planning methods a couple can use to DELAY becoming pregnant?*” and “*some of the permanent family planning methods a couple can use to AVOID children?*” Responses were then reviewed and coded as completely accurate (‘correct’) or not completely accurate (‘incorrect) for analysis. Related to abortion legality, participants were asked “*Is abortion legal in Nepal?*” and could respond with “Yes”, “No”, or “I don’t know”. Related to contraceptive ever use, newly married women and husbands were asked “*Have you ever used a contraceptive method?*” and could respond with “Yes”, “No”, or “I don’t know”. Related to pregnancy, newly married women were asked and open-ended question instructing them to quantify “*How many times have you been pregnant?*”.

Note that for some of the above measures, multiple survey responses were combined before analysis to understand summarize higher level shifts in family planning and contraceptive knowledge—specifically, identification of *all* family planning benefits as well as *all* temporary and *all* permanent contraceptive methods were analyzed as such. Additionally, responses on the sex preferences of children were analyzed as the binary report of desiring males and females as opposed to the desired number of each sex. Given the relatively small sample size by participant type, these strategies were more meaningful than approaches analyzing knowledge of a single specific benefit or method and more interpretable than differences between specific desired numbers of children of each sex.

### Data analysis

Quantitative data from the surveys were analyzed for changes in baseline and endline responses by participant type using Stata Statistical Software: Release 17 [31]. Following an assessment of normality using the Shapiro–Wilk Test, Wilcoxon Signed-Rank non-parametric tests—which requires six or more pairs and allows for unknown distribution—were conducted to compare sets of responses from the same participants and compute an exact p-value [32, 33]. Analysis by participant type was decided a priori to support understanding of differences by gender and generation. Specifically, we evaluated changes among survey responses among newly married women, their husbands, and mothers-in-laws separately.

The qualitative interviews were first transcribed and translated into English and then coded using Dedoose Version 9.0.17.[34] A codebook was developed by three team members (AM, AC and NDS), first by drawing on the interview guide and then by adding quotes that emerged in the analysis of a few interviews. The team iteratively developed the codebook and once the codebook was finalized, coding was done primarily by one team member (AC), with a subset of interviews being double coded (AM, NDS). Coded text was thematically analyzed, and results were compared by participant type to understand participant perspectives about the intervention’s impact on fertility and family planning norms and decision-making.

## Results

Participants included 90 individuals within triads across 30 households, evenly split between the Palinandan and Sunwal Municipalities of Nepal (Table 1). Among participants, newly married women had a mean age of 20.48 (SD: 1.83), husbands a mean age of 24.30 (SD: 3.92), and mothers-in-law a mean age of 50.10 (SD: 8.69). Approximately 75% of marriages between women and their husbands were described to be arranged, while the remaining were reported as “love” marriages. While most of the women (73.33%) and husbands (80.00%) reported achieving a high-school education or above, majority of mothers-in-law (86.67%) reported no formal education. Over 80% of all participants were Hindu.

**Table 1:** Participant demographic summaries

	Newly married women (n = 30)	Husbands (n = 30)	Mothers-in-law (n = 30)
**Mean age**	20.43 (SD: 1.83)	24.30 (SD: 3.92)	50.10 (SD: 8.69)
**Age range**	18 yrs–25 yrs	18 yrs–40 yrs	39 yrs–74 yrs
**Municipality**			
Palinandan (rural)	15 (50.00%)	15 (50.00%)	15 (50.00%)
Sunwal (peri urban)	15 (50.00%)	15 (50.00%)	15 (50.00%)
**Education**			
No formal education	2 (6.67%)	1 (3.33%)	26 (86.67%)
Elementary or middle school	5 (20.00%)	5 (16.67%)	4 (13.33%)
High school	22 (73.33%)	23 (76.67%)	0 (0.00%)
Beyond high school	0 (0.00%)	1 (3.33%)	0 (0.00%)
**Religion**			
Buddhist	1 (3.33%)	1 (3.33%)	1 (3.33%)
Christian	2 (6.67%)	2 (6.67%)	2 (6.67%)
Hindu	24 (80.00%)	24 (80.00%)	24 (80.00%)
Islam	3 (10.00%)	3 (10.00%)	3 (10.00%)
**Marriage type**			
Arranged	22 (73.33%)	22 (73.33%)	*NA*
Love	8 (25.81%)	8 (26.67%)	*NA*

Italics indicates findings were considered significant when p < 0.05 and highly significant when p < 0.01

### Impact on norms on fertility

#### Norms about timing of first pregnancy

Baseline and endline surveys revealed statistically significant changes to ideas about the timing of first pregnancy (Table 2).

**Table 2:** *Sumadhur* intervention impact on norms on fertility and on decision-making by participant type

			% Change pre- to post-intervention		
Norm category	Norm subcategory	Survey prompts & responses	Women	Husbands	Mother-in-laws
**Norms on Fertility**	*Timing of first pregnancy*	**It is wrong to use contraceptives or other means to avoid or delay pregnancy before having had at least 1 birth**	*p* = *0.015*	*p* = *0.017*	*p* = *0.085*
		Strongly agree	− 23.33	+ 20.00	− 16.67
		Agree	0.00	− 13.33	− 10.00
		Neutral	− 10.00	0.00	− 6.67
		Disagree	+ 23.33	− 6.67	+ 33.33
		Strongly disagree	+ 10.00	− 6.67	0.00
	*Spacing of pregnancies*	**It is wrong to use contraceptives or other means to avoid or delay pregnancy**	*p* = *0.009*	*p* < *0.001*	*p* = *0.031*
		Strongly agree	− 6.67	− 40.00	− 6.67
		Agree	− 6.67	− 6.67	− 3.33
		Neutral	− 6.67	0.00	− 10.00
		Disagree	− 3.33	− 16.67	+ 3.33
		Strongly disagree	+ 23.33	+ 56.66	+ 16.66
	*Norms on family size*	**How many children would you like to have?**	*p* = *0.453*	*p* = *0.663*	*p* = *0.453*
		One	− 6.67	0.00	0.00
		Two	+ 3.33	− 10.00	+ 13.34
		Three	+ 3.34	+ 3.33	− 13.34
	*Norms on sex preference of children*	**You desire at least one future child to be male**	*p* = *0.063*	*p* = *0.125*	*p* = *0.016*
		Yes	+ 10.00	− 3.33	+ 67.74
		No	+ 43.33	+ 26.67	+ 32.26
		Missing	− 53.33	− 23.33	− 25.81
		**You desire at least one future child to be female**	*p* = *0.063*	*p* = *0.375*	*p* = *0.016*
		Yes	+ 6.66	− 0.33	− 16.66
		No	+ 43.33	+ 23.34	+ 33.33
		Missing	− 50.00	− 23.33	− 16.67
**Norms on Decision− Making**	*Couple dynamics and decision-making*	**Most families you know believe men should make the decision about whether or not their wife uses birth control**	*p* = *0.383*	*p* = *0.754*	*p* = *0.125*
		Strongly agree	− 3.33	− 3.33	− 16.67
		Agree	+ 23.33	+ 6.67	+ 20.00
		Neutral	0.00	0.00	− 3.33
		Disagree	− 26.67	0.00	0.00
		Strongly disagree	− 13.33	− 10.00	0.00
	*Household roles and decision− making*	**Most families you know believe husbands should have the final say about when to start trying to have their first child**	*p* = *0.163*	*p* = *0.629*	*p* = *0.144*
		Strongly agree	− 10.00	− 3.33	− 16.67
		Agree	+ 50.00	+ 13.33	+ 46.67
		Neutral	− 30.00	0.00	− 3.34
		Disagree	0.00	0.00	0.00
		Strongly disagree	− 10.00	− 16.67	− 26.66
		**Most families you know believe husbands should make the final decision about the total number of children they want**	*p* = *0.048*	*p* = *1.000*	*p* = *0.263*
		Strongly agree	− 10.00	0.00	− 16.67
		Agree	+ 56.66	− 6.66	+ 43.33
		Neutral	− 26.67	0.00	0.00
		Disagree	0.00	0.00	0.00
		Strongly disagree	− 20.00	0.00	− 26.67
	*Individual intentions*	**Do you think you will use a contraceptive method to delay or avoid pregnancy at any time in the future?**	*p* = *0.008*	*p* = *0.125*	*N/A*
		No	0.00	− 6.67	–
		Yes	+ 26.66	+ 10.00	–
		Unsure	− 26.66	− 10.00	–

Italics indicates findings were considered significant when p < 0.05 and highly significant when p < 0.01

### Impact on Norms on Fertility

At baseline, 70% of participants agreed or strongly agreed that “it is wrong to use contraceptives or other means to avoid or delay pregnancy before having had at least one birth”, and this decreased to 57% at endline. The change was most significant among newly married women (*p* = 0.015). Interestingly, shifts were reported in opposing directions by gender. While 23% fewer newly married women and 17% fewer mothers-in-law strongly agreed that “it is wrong to use contraceptives or other means to avoid or delay pregnancy before having had at least one birth” at endline, 20% more husbands strongly agreed compared to baseline.

In general, qualitative findings underscored the strength of societal pressures to prove fertility early in marriage. When asked about the consequences of delaying the first pregnancy and birth, most participants acknowledged deeply ingrained societal expectations of birth within the first year of marriage though often personally disagreed with this pressure. The separation of personal and societal beliefs was common across participants, many of whom noted the historical norms of early childbirth but did not prescribe to this view themselves. Even within interviews, participants wrestled with the perceived harms and benefits of obliging with social fertility norms to conceive early in marriage. While delaying birth was seen to lead to safer outcomes for young women and increased stability for the infant, early conception squelched community gossip and stigma and increased community belonging. For example, one mother-in-law explained that, for her, educational and employment goals in addition to young maternal age were reasonable justifications to delay first birth. However, she went on to acknowledge the sometimes-unbearable weight of societal stigma, compounded by the positive reinforcement and community embrace that occurs following a first birth.

Families also frequently described navigating long-held beliefs that using contraception to delay a first pregnancy could lead to infertility. Even though these beliefs were considered “old thoughts” and “an outcome of illiteracy,” it was clear that they continued to influence fertility decision-making among the younger generations. For several couples, this limited the types of contraception they were willing to use—often relying only on male condoms. Early first pregnancy norms were further affirmed by pregnancy findings among newly married women and over the course of the four-month intervention, 37% of newly married women reported becoming pregnant.

Despite persistent norms to prove fertility early, several families spoke affirmatively towards delaying the first birth following their participation in *Sumadhur*. Some families noted that the intervention evoked familial and community conversations conveying both recognition of the norms shaping the older generation alongside a willingness to support the changing priorities of the younger generation. One mother-in-law summarized that while “Everyone uses the family planning measures only after having a first child”, she knows and supports that her “daughter-in-law doesn't have any desire to be a mother this soon as she wants to have a baby after completing her studies” (58-year-old Mother-in-Law in family triad #9, Palhinandan). The daughter-in-law in this triad recognized this shift in her mother-in-law’s opinion and described her surprise on this:

“My mother-in-law had a very old way of thinking. She used to tell me to plan for childbirth right after marriage. But after participating in the program, she now tells me to have a baby only after completing my studies and getting a good job. I’m very happy to see my mother-in-law change completely. She even helps me in the household work, and talks to me very nicely giving her suggestions.” (21-year-old Newly Married Woman in family triad #9, Palhinandan).

This sentiment was shared by other families as well. When asked what surprised her about the intervention, one mother-in-law admitted: “Me and my son used to say to my daughter-in-law that we need a baby soon, but now the things have changed. My son and daughter-in-law are not in hurry. I have also realized that I shouldn’t pressure them. They are still young… I am happy with their decision to delay first childbirth.” (41-year-old Mother-in-Law in family triad #3, Palhinandan).

Participants’ perception of *Sumadhur’s* success also led to reflections about the benefit this knowledge could have *prior* to marriage. Couples in particular explained that effective prevention of early pregnancy would require intervention before a couple is married given societal pressure for immediate pregnancy. When asked whether others would benefit from this intervention, one newly married woman summarized:

“It will be good if you can keep other newlywed couples like us. But it will be better if you bring the unmarried ones to participate; if they get knowledge on these things before getting married, they will know what to do after marriage. We would have known more if we had got a chance to participate in this program before marriage, we would have known when to plan for a baby and what should be the right year gap for another child. I didn’t know anything, so I am pregnant already.” (said laughingly) (23-year-old Newly Married Woman in family triad #16, Sunwal)

Similarly, another newly married woman shared that the only thing she could not implement from the training was her desire to use contraception to delay pregnancy in order to pursue higher education, because she became pregnant immediately after marriage and prior to participation in the intervention. She went on to share that the information she had gained about child spacing would allow her to complete education between births, however, she also recommended engagement earlier among women in her community:

“These programs should be implemented before we get pregnant. In our area, women get pregnant immediately after marriage and such trainings if provided at the preconception period might help ambitious people like us and cooperative households like ours. Apart from that, such education should be given in school. Also, people should know about this before they get married.” (21-year-old Newly Married Woman in family triad #13, Palhinandan).

Husbands agreed with this, acknowledging competing desires to pursue personal advancement via employment or education and appeasing familial or community expectations. One husband shared that while he did not regret that his wife was already pregnant, he wished they could have used a contraceptive method to delay pregnancy. When asked specifically if it would be acceptable to use contraception prior to a first birth, he advocated: “Yes, they should use the method. This is because if they use it, they can prevent pregnancy. If they want to work, they can continue working. They can use it for certain time and stop it if they want a baby.” (30-year-old Husband in family triad #23, Sunwal). He went on to explain that while relatives and community members are allowed to provide advice to couples, their opinions shouldn’t be binding, explaining “I don’t think they should force anyone to give birth. It is completely husband and wife's decision. We shouldn’t be under someone's influence about giving birth… But I am okay if they give advice or suggestions.”

#### Norms about the spacing of pregnancies

Across all participant types, significant changes were observed related to norms about the spacing of pregnancies. All participants trended significantly toward strongly disagreeing or disagreeing that it is wrong to use contraceptives or other means to avoid or delay pregnancy—29% agreed it was wrong to use contraception to space pregnancies at baseline, which decreased to 6% at endline (Table 2). The most dramatic shift (*p* < 0.001) was seen among husbands as 53% agreed it was wrong pre-intervention which decreased to 7% at endline. Qualitatively, all participants supported contraceptive use as a means of pregnancy prevention following the first birth. Several couples described both increased knowledge and increased communication between husband and wife because of participation in the intervention, strongly illustrated by one newly married woman’s response to changes in views following the intervention:

“There has been changes in many of my views. Nowadays I give due respect to my husband and mother-in-law. And they reciprocate the same feeling by loving me and respecting me. I did not know anything about the use of family planning methods earlier, but now I feel I’m fully aware about the topic. Well, my current pregnancy happened because of my lack of awareness. But I will bear another child three to four years after of birth of my first child…Actually, me and my husband have discussed about using this. Earlier, there used to be no discussion between me and my husband regarding the number of children to bear as a couple and use of family planning methods; but now we discuss about these topics in detail.” (21-year-old Newly Married Woman in family triad #8, Palhinandan)

All participant types reflected positively about family planning knowledge, naming its relevance between pregnancies. These findings correlated with significant increases in knowledge related to birth spacing benefits across all participants. At endline, an additional 12% of newly married women, 60% of husbands, and 17% of mothers-in-law identified at least one benefit of birth spacing (Table 2). The most reported benefits at endline included better health for mothers and children as well as better control of socioeconomic status and personal expenses.

#### Impact on norms on family size

Significant changes were not observed for all participant types related to norms about family size. Across types, participants generally tended to report wanting one or more additional children at endline compared to baseline (Table 2). While qualitative reflections on this topic were limited, there was little indication of strong societal pressure or influence to have a certain number of children. Generally, participants saw this as a decision for couples to make. One mother-in-law explained, “And regarding the number of children, it is better that they are decided as per the wish of the husband and wife instead of other people’s sayings.” (61-year-old Mother-in-law in family triad #14, Palhinandan). Additionally, while couples frequently suggested their family size was unpredictable, they also reported specific desires. In response to how many children she wanted to have, one newly married woman explained “Whatever happens, but I will bear two babies in total (she said this with a smile).” (23-year-old Newly Married Woman in family triad #29, Sunwal). At endline, most of all participants desired two children or grandchildren.

#### Impact on norms on sex preference of children

Although t-tests revealed significant changes to norms on sex preference of children, quantitative findings were difficult to interpret as sex preference questions within the pre and post surveys were dependent upon answers to questions about family size preference and participants generally reported desiring more children (Table 2). Across all participants at endline, significantly fewer participants specified desiring male children or female children suggesting an overall increased openness to either sex. This trend is strongly supported by qualitative findings and appears to be a generational shift. One mother-in-law summarized: “Well, people do not give birth to more than two children. It does not matter if it’s son or daughter, two children are enough. In our time, we used to have babies until son is born. My mom too gave birth to five daughters before having a son. Today's daughters-in-law do not wait to have son. They just have two children whether both are boys or girls.” (45-year-old Mother-in-law in family triad #28, Sunwal).

Newly married women sensed this shift as well and one suggested that family members talked more openly after the intervention and expressed love toward anticipated children of all sexes: “Many family members don't show anger these days while talking. They knew about many things after the training which helped in changing their behavior… family members used to despise women if they give birth to a daughter. They don't do such these days. They love son and daughter equally.” (21-year-old Newly Married Woman in family triad #13, Palhinandan).

### Impact on norms on decision-making

#### Norms around household dynamics and decision-making

Most participants across all types agreed that “most families they know believe men should make the decision about whether or not their wife uses birth control” at both baseline and endline (Table 2). Across all participant types, no strong changes were seen related to the belief that “most families they know believe husbands should have the final say about when to start trying to have their first child” and everyone generally agreed this was true at both baseline and endline. Similarly, a lack of significant change among newly married women and husbands was observed related to the norm that most families believe husbands should make the final decision about the total number of children they want. Statistically significant change was observed among newly married women. Interestingly, there was both a decrease in the percentage of mothers-in-law that strongly agreed with this statement and an increase in the percentage of mothers-in-laws who agreed that most families believe husbands should make the final decision about the total number of children they want. While qualitatively few participants spoke specifically about fertility decision-making roles, those that did suggest it should be something couples agree upon and decide together.

Qualitative findings suggested that in some cases, household dynamics shifted in the absence of early pregnancy in that it increased the risk of violence between couples. Unsurprisingly, the fear of negative dynamics was described to motivate some couple’s decisions to seek pregnancy early after marriage. One newly married woman explained:

“After getting married, everyone said that it will be good if I get pregnant right away. I was told that my sister-in-law couldn’t get pregnant for three years post her marriage. This caused a problem in her relationship with her husband, and he used to scold and beat her. My mother-in-law was scared that it might happen to me too, so she suggested me to get pregnant early… that’s why we did it.” (23-year-old Newly Married Woman in family triad #16, Sunwal).

Another newly married woman described additional possible consequences including infidelity and humiliation as harmful to couple dynamics:

“There can be quarrel at the home, and it is likely that the husband might start seeing another woman… not all male partners behave that way, some are very understanding. But even while the husband is understanding, the people in the community might directly tell the husband that “your wife is unable to bear a baby”, which makes husband feel bad and humiliated. Because of this, there can be heated arguments between the couple and can negatively impact their marital relationship.” (23-year-old Newly Married Woman in family triad #29, Sunwal).

Quantitative findings related to household roles and decision-making were nuanced as participants described gradual shifts toward increased gender equity related to household decision-making. Similar to societal pressures related to early first birth, many participants alluded to historically strong beliefs about gendered household roles while acknowledging the benefits of challenging these norms. One husband described the woman’s role by explaining that “The majority of women in our region are limited to kitchen. But they should be given opportunity to come outside their household. They need to be educated, aware, and should get the platform to work and earn money. Similarly, they also need to take part in household conversation as well as decision making. They can also lead if given the platform to lead.” (26-year-old Husband in family triad #9, Palhinandan). Another husband seemed to desire to express women’s autonomy though immediately recalled a limiting societal norm:

“There are three women in my family and two males. We all have our specific roles. We do not restrict women. My wife is working, and we do not restrict her. We don’t allow menstruating woman to enter the kitchen at our home and my wife complains about this as she is against this. Apart from that there are no restrictions.” (23-year-old Husband in family triad #19, Sunwal).

Following the intervention, participants consistently expressed recognition of and desires for increased women’s empowerment:

“I think women should take a part in decision making. They can also contribute to the family through many ways. Some might work outside home whereas others might do all the household chores. Everyone should be respected and treated well. We should listen to female members of the family and take their opinion in any important matter.” (23-year-old Husband in family triad #28, Sunwal)

“In earlier days, people used to say that daughter-in-law is not a core family member. She doesn’t do anything for the family and only looks after her own benefit. But nowadays, people say that son and daughter-in-law are equal. They support and respect them as well. The belief system as well as what they practice both has changed in recent times. Discrimination between daughter and daughter-in-law has decreased gradually. It feels good seeing all these changes.” (21-year-old Newly Married Woman in family triad #13, Palhinandan)

“Earlier, women were not valued, and their opinion was not asked on any matters. All the decisions were made by male household head. But these days, when decisions are to be made, they ask for the suggestions of the women of the family too. They are also involved in the household decisions. It seems to be good to involve everyone’s suggestions in the household decision making.” (54-year-old Mother-in-law in family triad #9, Palhinandan)

Qualitative interviews suggested that *Sumadhur* improved couple dynamics by facilitating increased and intentional conversations that allowed couples to engage in shared decision-making.

#### Individual intentions

While one third of newly married women were unsure about whether they would use a contraceptive method to delay or avoid pregnancy at any time in the future at baseline, 93% reported they would at endline which was an increase of 27% for these participants (Table 2). Among husbands, intention to use a contraceptive method in the future increased by 10%, to 90% at endline. Throughout qualitative interviews, participants reported starting or continuing contraceptive methods as a result of the intervention. Some couples described that they were motivated to establish additional economic security prior to having children and others suggested that they were glad to have additional knowledge about how to space their planned pregnancies. For one husband, increased information about the potential side effects of female contraception seemed to influence his own decision-making. He explained, “Yes, I already knew about family planning methods, but I hadn’t used it. I came to know about different family planning methods that can be used by female. I didn’t know in detail regarding this previously. I feel that men should use condom as other methods are focused on women and have negative impacts.” (27-year-old Husband in family triad #27, Sunwal).

These findings aligned with shifts in ever use of contraception behaviors for both newly married women and husbands. Among newly married women, an additional 13% reported ever use at endline compared to baseline. Similarly, an additional 16% husbands reported of ever using contraceptives in endline compared to the baseline. However, the changes were not statistically significant, perhaps due to small sample sizes because some newly married women were already pregnant (Table 2).

In response to whether she had changed her thinking about contraceptives as a result of *Sumadhur*, one newly married woman reflected:

“Everyone was putting their thoughts into the discussion... I feel that I want to have a child only after three years from now. I’m just 19 years old and I am too small to be a mother. Therefore, I don’t want to have a baby now. I want to have my child after three years. And I want only one child. That’s why I’m taking pills these days.” (19-year-old Newly Married Woman in family triad #1, Palhinandan).

This woman went on to say “They never used to involve me in any household matters earlier. Now, they also ask me for my opinions in any matters and take my suggestions too.” These findings seem to support the idea that increasing women’s empowerment can increase the likelihood they have the agency to act upon their individual intentions, including those related to fertility and family planning.

### Knowledge and behavior

Additional changes in knowledge related to family planning and abortion was found across participants (Table 3).

**Table 3:** Change in family planning knowledge and behavior by participant type

	% change baseline to endline					
	Women (%)	p-value	Husbands* (%)	p-value	Mothers-in-law (%)	p-value
Identifies 1 or more benefits of family planning/birth spacing	+ 20.00	0.031	+ 60.00	< 0.001	+ 16.67	0.125
Identifies correct family planning methods to temporarily delay pregnancy	+ 10.00	0.250	+ 6.67	0.063	+ 20.00	0.063
Identifies correct family planning methods to permanently prevent pregnancy	+ 33.34	0.001	+ 50.00	0.002	+ 20.00	0.031
Responds correctly (“Yes”) to ‘*Is abortion legal in Nepal?*’	+ 70.00	< 0.001	+ 40.00	< 0.001	+ 67.67	< 0.001
Reports ever use of a contraceptive method	+ 13.33	0.180	+ 16.66	0.227	*NA*	*NA*
Reports 1 or more pregnancies	+ 36.67	0.500	*NA*	*NA*	*NA*	*NA*

Italics indicates findings were considered significant when p < 0.05 and highly significant when p < 0.01

*post intervention data was missing for 2 husbands

**NA = question not asked of participant type

All participant types improved their ability to correctly identify both temporary and permanent family planning methods; pre-post change was marginally significant (*p* = 0.063) for mothers-in-law’s and husband’s knowledge of temporary methods, and statistically significant for all groups related to permanent methods (*p* < 0.05). Participants discussed specific myths dispelled by the intervention including the belief that condoms eliminate pleasure or that IUDs and pills often cause uterine harm. In the qualitative interviews, they also openly discussed their decision to begin a method. When asked about her changed opinions, one newly married woman summarized:

“We have been using condoms these days to plan for a baby later... Earlier, we, husband and wife never used to talk openly but it has changed now. We talk openly with each other. Love and care have increased between us. We are thinking of having a child after my husband gets a job. My in-laws also say the same.” (20-year-old Newly Married Woman in family triad #5, Palhinandan).

Several participants expressed gratitude for their changed knowledge and behavior summarized by one husband’s comments, “I think the training was fruitful. I learned many things from the training. I started using family planning method and we all eat together nowadays. So, I am very thankful for the trainers and people like you who are trying to make our lives better.” (19-year-old Husband in family triad #25, Sunwal).

Participants also acknowledged that even when knowledge had changed, additional barriers remained that may influence behavior. For example, when asked what sort of barriers couples may face to using family planning methods, one mother-in-law explained “They might fear or feel shy. They might not get the device of their choice. Some might have problem to access the device as health post might be located far away.” (48-year-old Mother-in-law in family triad #13, Palhinandan).

Dramatic improvements were also observed across participant types related to knowledge of the legality of abortion in Nepal. Compared to baseline, an additional 70% of newly married women, 40% of husbands, and 67% of mothers-in-law correctly responded yes to the question “Is abortion legal in Nepal?” (*p* < 0.001) (Table 3). While no participants reported a personal experience of undergoing an abortion at baseline or endline, increased awareness was supported by qualitative findings as well. One husband shared:

“I didn’t know much about abortion. After the training, I came to know that abortion can be done up to 12 weeks of pregnancy. It can be done if both husband and wife don’t have a desire for a baby. It can also be done in case of health risk of mother as well as her baby. Also, they said it is free of cost.” (24-year-old Husband in family triad #5, Palhinandan)

## Discussion

Our findings suggest that *Sumadhur* impacted social norms and determinants of fertility and family planning knowledge and behaviors among newly married women, their husbands, and mothers-in-law in Nepal. Specifically, norms around timing, spacing, and sex preference of children changed among our population after the intervention, as did individual level knowledge about family planning and abortion, intentions to use family planning and actual family planning use. The increase in knowledge about contraception, and related individual intentions and use are likely due in part to the fact that participants had very low levels of knowledge and little exposure to these topics among this population prior to the intervention. The shift in norms around the timing, spacing and sex preference were exciting to see, as these have previously been seen as entrenched norms that were hard to shift, especially around sex preference and the timing of the first pregnancy.

Norms around decision-making, specifically the importance of the role of men as the primary decision-maker, were harder to change. It appears that gendered norms about the husband’s role in decision-making were stronger in our sample than has been found in some other samples in Nepal, where it seemed there was less emphasis on the need for men to be the primary decision-maker [27]. One recent study found a similar pattern of persistent, restrictive gender norms despite ongoing efforts and continued social change [35]. These findings suggest ongoing research is needed to identify effective strategies to shift gender norms. In our study, respondents of all types described changing roles throughout the qualitative findings, describing that family members helping more in the house and newly married women being given more opportunities to participate. Thus, it is possible that women’s agency did increase in some ways through the intervention, however, the social norms around men’s role as decision-makers still may have resulted in respondents stating that men should be the primary decision-makers. If indeed there were shifts in some behaviors around household roles and decision-making, these are likely to eventually have downstream effects on women and children’s health outcomes. Similar to the decision-making norms, norms around the desired family size also did not shift, but this may be due to the fact that desired family sizes are already quite small in South Asia, including Nepal, where the total fertility rate already hovers at 2.3 children per woman [5].

It is important to note that while we did see change in norms around the timing of the first birth, this was not in the direction we hoped for in the case of the husbands, and across all groups there still were sizeable proportions of respondents who did not agree with couples being allowed to delay the first birth with contraception. Our findings about newly married husbands warrant further exploration, and the need to disentangle whether there was some aspect of the intervention which had the unintended response. However, overall, the qualitative interviews shed much light onto the strength of the social norm to prove fertility early in marriage—for women’s position in the household, family “harmony”, and to avoid gossip about her infertility. Understanding more about competing influencers on this norm is critical—especially because the qualitative data suggested that for the most part respondents seemed to have a desire to delay the first birth. While there is some recent literature which describes women in South Asia having an interest in delaying the first birth, the vast majority of research and interventions are still rooted in the belief that the cultural norms around timing of the first birth are too steadfast to change [36].

An ongoing theme was the tension that participants felt between their own shifting norms and entrenched societal norms that they perceived not to be shifting. Repeatedly, respondents discussed how society was not progressing, and they described traditional norms. At the same time, respondents discussed that they themselves did not adhere to these expectations and that the circumstances were different in their own families. This was especially true for norms around timing of the first birth, but also emerged for other norms. Limited previous literature has described this tension, including in formative work from this same study population [37]. The current study highlights support for changing social and gender norms around fertility from across the generations (mothers-in-law as well as young couples) and across sexes (men and women). One goal in the design of our intervention was that by bringing groups of households together, we could address both individual norms (what people say that they believe themselves) as well as injunctive norms (what individuals think other people think they should be doing). Previous studies from Nepal had highlighted the influence of common behaviors in one’s community on predicting future desires and decisions, such as timing of the first birth [38]. For example, we hoped that when participants in our study—most of whom personally believed it was acceptable to delay the first birth—heard others in their own communities also believe it is okay to wait, that this would reinforce the acceptability of shifting expectations. However, the qualitative interviews suggested that shifting these perceptions about what other think (social norms) might be more challenging and require other approaches. We found that while generating aligned understanding of community norms was important, translating change to collective behaviors will require continued efforts. In this way, our findings support the idea that empowerment is insufficient without increased support for locally-driven educational, vocational, political, and economic opportunities for women which support the national constitution’s proclamation of equality for women [39]. Ensuring and advancing agency will likely require increased participation of women across the lifespan, supporting the myriad of societal assets they offer beyond childbearing.

Alongside norms, knowledge and behaviors showed significant shifts. Most notably, all participants’ knowledge regarding the legality of abortion improved. This is critical as according to Nepal’s 2016 Demographic and Health Survey, only 41% of women aged 15–49 were aware of the legality of abortion, and just 23% knew that abortion can be obtained up to 12 weeks for any reason[40]. Further, of those who knew that abortion is legal, less than half (48%) knew where to obtain safe services [40]. Prior literature has contextualized the challenges that young Nepalese women often face if they experience an unplanned pregnancy due to patriarchal pressures and expectations [41]. Misconceptions about abortion laws obscures health care access and increases the risk of unsafe abortion which can have negative short- and long-term health outcomes [41]. Lack of awareness about the legal provisions for abortion and about the availability, location and costs of services continues to prevent many women from accessing safe and legal abortion services in Nepal [6, 10]. Additional literature suggests that improving provider knowledge will be an additional critical step to increasing access [42]. Qualitative findings confirm that reducing barriers, particularly to early abortion, for women in Nepal continues to be important related to women’s autonomy and agency over their fertility and health [43]. An improved understanding of the scope and application of current abortion law may facilitate increased access to services that are already available.

Participants strongly recommended the continuation and expansion of *Sumadhur*, suggesting it would be sustainable and particularly impactful among rural and marginalized groups. Longitudinal research may be a beneficial next step to assess the sustainability of the intervention’s impacts. In addition to the intervention’s feasibility and acceptability, significant changes were immediately observed across a myriad of deeply rooted social norms surrounding fertility and family planning for newly married women, husbands, and mothers-in-law. The results demonstrate the success of the intervention in advancing gender equity and empowerment, particularly among women and mothers-in-law—finding ways to engage husbands, and, potentially fathers-in-law, is key for future researchers. The powerful and positive impact that *Sumadhur* had on fertility and family planning norms, knowledge, and behavioral intentions suggests it should be scaled up and replicated for implementation in similar settings across Nepal and globally.

## Limitations

While this study has many strengths, including its mixed-methods approach and data collection from household triads, it has a few limitations. First, this is a pilot study, and was only conducted in two municipalities of one district, of Nepal. Thus, the sample size is fairly small (for the quantitative survey) and findings may not be generalizable to other parts of Nepal or elsewhere, which may have different social norms related to these topics. Nevertheless, the social norms in this sample are similar to those described in Nepal and other South Asian countries.[1, 3, 5, 6] Second, this intervention was conducted during the COVID-19 pandemic. The intervention took place right before the biggest surge in the COVID-19 pandemic that Nepal experienced, and thus qualitative interviews were delayed until about 3 months post-intervention. The COVID-19 pandemic may have impacted people’s experiences participating, however, did not appear to impact participation itself. The pandemic may also have impacted fertility preferences, household dynamics, experiences of violence including intimate partner violence, and other factors associated with this study, as has been found elsewhere [44]. The directionality that the pandemic might have had on these norms and behaviors and if it would have heightened or muted the impact of the intervention is unclear. Nevertheless, the highly promising findings across areas of impact suggest the intervention may be beneficial regardless of pandemic status. Finally, our construction of the question about sex preference of future children made it difficult to precisely measure the change in this outcome, making data related to sex preference unclear. Because there was an overall increase in desired number of children and the question wording asked for quantity of boys/girls/either, there was no statistical significance. However, qualitative data seems to suggest important shifts, dismantling historical preference for male children occurred.

## Conclusion

Perceived, unwritten societal expectations about the timing and spacing of pregnancies, norms on family size and sex preference of children, gender norms, and household roles and couple dynamics influence health outcomes across the lifespan. Prevention of early and closely spaced pregnancies can mitigate gender inequality by protecting women’s opportunities to continue education and/or participate in the workforce or give them time to adjust to living in a new household. This study underscores the importance of engaging influential family members, including mothers-in-law, to successfully shift household decision-making and impact long-held norms. Improving household and couple relationships facilitates reproductive autonomy and personal agency by strengthening communication and understanding surrounding the choices couples make related to fertility and family planning. The public and women’s health strategies of the Ministry of Health & Population of Nepal would be strengthened by the inclusion of a pre-pregnancy intervention with an emphasis on gender norms and social change for newly married couples and their families. Achieving improved health outcomes among newly married women is critical to promoting health across the lifespan. Promoting agency ensures that communities can thrive by empowering its members with the knowledge and resources needed to implement behaviors that advance health and wellbeing.

## Data Availability

Data used for this study are available upon request from the authors.
